# Unusual Presentation of COVID Pneumonia as Esophageal Rupture Ended With Successful Management

**DOI:** 10.7759/cureus.17348

**Published:** 2021-08-21

**Authors:** Ali Rahman, Sura Alqaisi, Chad Downing

**Affiliations:** 1 Internal Medicine, Northwell Health at Mather Hospital, Port Jefferson, USA; 2 Radiology, Stony Brook University, Port Jefferson, USA

**Keywords:** boerhaave's syndrome, complex pleural effusion, coronavirus disease 2019 (covid-19), esophagogram, mallory-weiss tear

## Abstract

Coronavirus disease 2019 (COVID-19), caused by severe acute respiratory syndrome coronavirus 2 (SARS-CoV-2), is a severe respiratory syndrome. It started as an epidemic in Wuhan, China, and then become a global pandemic. COVID-19 usually presents with respiratory symptoms, including cough and shortness of breath, accompanied by fever. However, gastrointestinal symptoms, such as nausea, vomiting, and abdominal pain, have also been reported as a less common presentation of COVID-19. Boerhaave syndrome is a transmural perforation of the esophagus that typically occurs after forceful emesis, which should be differentiated from Mallory-Weiss syndrome, a nontransmural esophageal tear. Diagnosis of Boerhaave syndrome can be difficult because of the classic symptoms, resulting in a delay in seeking medical care. Boerhaave syndrome is sporadic, with an incidence of 3.1 per 1,000,000 per year.

We present an interesting case of a 53-year-old man who presented to the emergency department (ED) complaining of significant right-sided chest pain and diffused abdominal pain after several episodes of coughing and vomiting associated with shortness of breath and fever for two weeks. The patient was found to have COVID- 19 infection. The patient then had a chest CT without contrast, and an esophagogram was performed, which was consistent with esophageal rupture. The patient had a thoracotomy and surgical repair. This was followed by endoscopy and esophageal stent placement.

The COVID-19 pandemic is a major health crisis that has drained medical resources and research capacity. Esophageal rupture is commonly due to iatrogenic causes. Transmural perforation following forceful vomiting has been termed Boerhaave syndrome. Often, it has no specific presentation, which can lead to late diagnosis, delayed treatment, and increased mortality. In this case, the early diagnosis and proper implementation of the general principles of treatment, including sepsis control, drainage, and surgical repair, led to a good outcome for the patient.

## Introduction

Coronavirus disease 2019 (COVID-19) caused by severe acute respiratory syndrome coronavirus 2 (SARS-CoV-2) is a severe respiratory syndrome that has negatively impacted the whole world over the past year. It started as an epidemic in Wuhan, China, and then became a global pandemic. COVID- 19 usually presents with respiratory symptoms, including cough and shortness of breath with a fever. However, gastrointestinal symptoms, such as nausea, vomiting, and abdominal pain, have also been reported as another presentation of COVID-19 [1]. The Centers for Disease Control and Prevention (CDC) estimates that 2.5% of the United States population has already been infected with COVID-19 [2-3]. Physicians should broaden their differential diagnosis list regarding the clinical presentations and complications that patients with COVID-19 may show.

Boerhaave syndrome is a transmural perforation of the esophagus that typically occurs after forceful emesis. It should be differentiated from Mallory-Weiss syndrome, a nontransmural esophageal tear [4]. Diagnosis of Boerhaave syndrome can be difficult because, often, no classic symptoms are present and delays in presentation for medical care are common. Although Boerhaave syndrome classically presents as the Mackler triad of chest pain, vomiting, and subcutaneous emphysema due to esophageal rupture, these symptoms and signs are not always present, then on imaging of Esophagogram with oral contrast, usually shows the leaking of the contrast material in the mediastinum, or pleural effusion mostly on the left side more than the right, and on pleural fluid analysis, one of the most common criteria is to have a very low ph due to gastric acidity [4].

Management of Boerhaave syndrome usually includes sepsis control with broad-spectrum antibiotics, nasogastric tube suction, parenteral nutrition, tube thoracostomy, surgical repair of the perforation with the possibility of esophageal stenting, and endoscopic suture ligation [4].

Boerhaave syndrome is very rare, with an incidence of 3.1 per 1,000,000 per year. Among esophageal perforations, approximately 15% are spontaneous perforations [5]. We present an interesting case of a 53-year-old man who presented to the ED with a chest and abdominal pain complaint after having multiple episodes of coughing and vomiting. He was found to have COVID pneumonia and Boerhaave esophageal rupture. After successful treatment, he became one of the few survivors of esophageal rupture.

## Case presentation

A 53-year-old man presented to the ED complaining of significant right-sided chest pain and diffuse abdominal pain after several episodes of coughing and vomiting associated with shortness of breath. His symptoms started several hours earlier while he was watching TV at home. He reports having shortness of breath, coughing, vomiting, and fever for two weeks. They got progressively worse, which prompted him to come to the ED. His past medical history was significant for coronary artery disease, diabetes mellitus, remote alcohol abuse (quit alcohol 15 years ago), chronic pancreatitis, and chronic kidney disease. He is an active smoker, half a pack per day, and lives alone. He denies substance abuse.

In the ED, the patient was found to be febrile, tachycardic, hypotensive, and tachypneic. His oxygen saturation (SpO_2_) was 94% on 4L O_2_ via nasal cannula. On examination, he looked ill, was in acute distress, had markedly reduced breath sounds on the right lower side of his chest. His abdomen was distended with diffuse tenderness, and bowel sounds were active. His laboratory results (Table 1) were significant for leukocytosis, and he was SARS-CoV-2 polymerase chain reaction (PCR) positive.

**Table 1:** Patient laboratory results in the ED Hgb: hemoglobin; PLT: platelet; BUN: blood urea nitrogen; APTT: activated partial thromboplastin time; PT: prothrombin time; SARS-CoV-2: severe acute respiratory syndrome coronavirus 2

Labs	Results	Normal Range
WBC	16.4 * 10^9/L	4.5 -11 *10^9/L
Hgb	15.5 g/dl	13.4 - 17.4 g/dl
PLT	307*10^9/L	150 - 440*10^9/L
Lipase	<6 U/L	4 - 25 U/L
Troponin	0.1 ng/ml	0 - 0.5 ng/ml
Na	136 mEq/l	134 -146 mEq/l
K	3.5 mEq/l	3.5 -5.1 mEq/l
Cl	91 mEq/l	92 -109 mEq/l
CO_2_	18 mEq/l	24 - 31 mEq/l
Glucose	198 mg/dl	60 -100 mg/dl
BUN	37 mEq/l	8 - 25 mEq/l
Creatinine	1.46 mEq/l	0.5 - 1.5 mEq/l
APTT	28 sec	21.5 - 31.9 sec
PT	12 sec	10 - 13 sec
SARS-CoV-2	Positive	Negative
Lactate	3.1 mg/dl	4 -16 mg/dl

**Figure 1:**
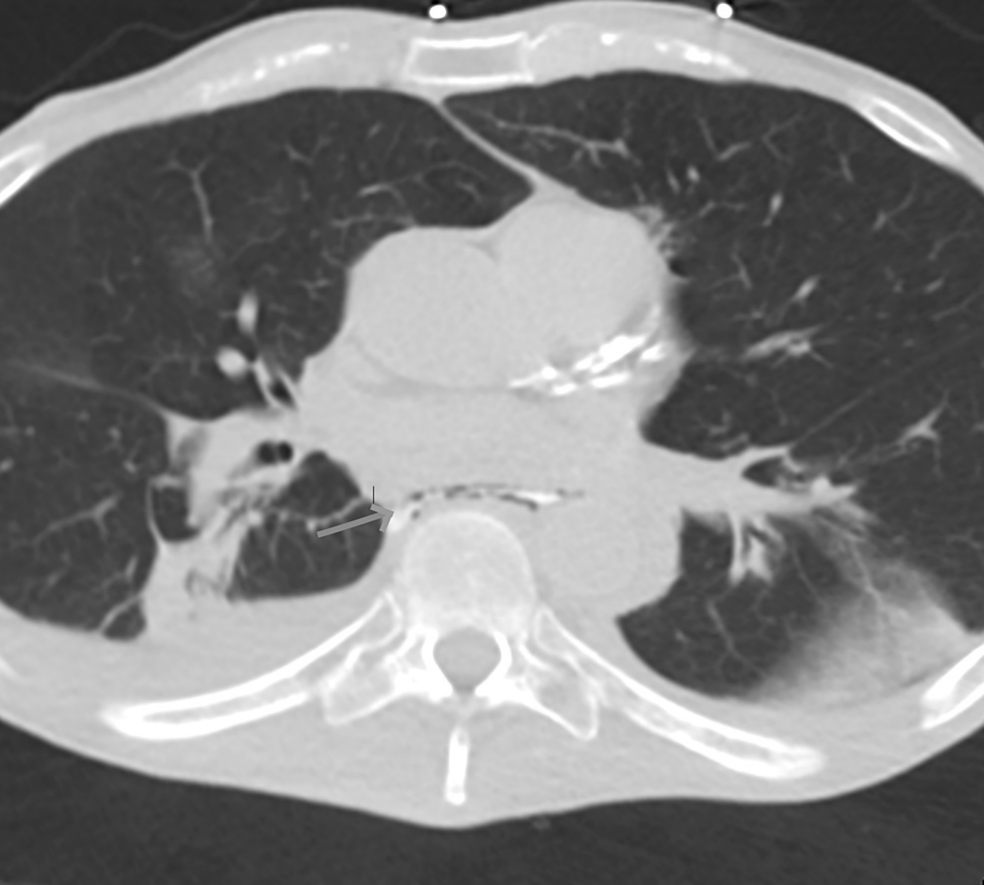
This axial computed tomography image demonstrates pneumomediastinum and free flow of the enteric contrast material into the mediastinum outside the esophageal lumen compatible with esophageal tear

His electrocardiogram (EKG) showed sinus tachycardia with no significant ST-T wave changes. A computed tomography (CT) chest scan with oral contrast (Figure 1) showed a pneumomediastinum suggesting Boerhaave syndrome, a tiny right hydropneumothorax, and right lower lobe segmental atelectasis secondary to mucus plugging.

The ED consulted the thoracic surgery department, and a chest tube was placed on the right side, which drained approximately 800 cm3 dark brown thin fluid, which was sent for analysis. The pleural fluid analysis (Table 2) showed highly acidic exudative fluid. 

**Table 2:** Pleural fluid analysis LDH: lactate dehydrogenase

Pleural fluid parameters (Units)	Values (Normal Range)
Color	Amber (Clear)
PH	6 (7.60-7.64)
WBC (cell/ml)	22260 (<1000)
Glucose (mg/dl)	89 (60-100)
Protein (g/dl)	4.1 (1-2)
LDH (U/l)	4989 (<250)
Amylase (U/l)	10605(30-110)
Triglycerides (mg/dl)	33(<50)

**Figure 2:**
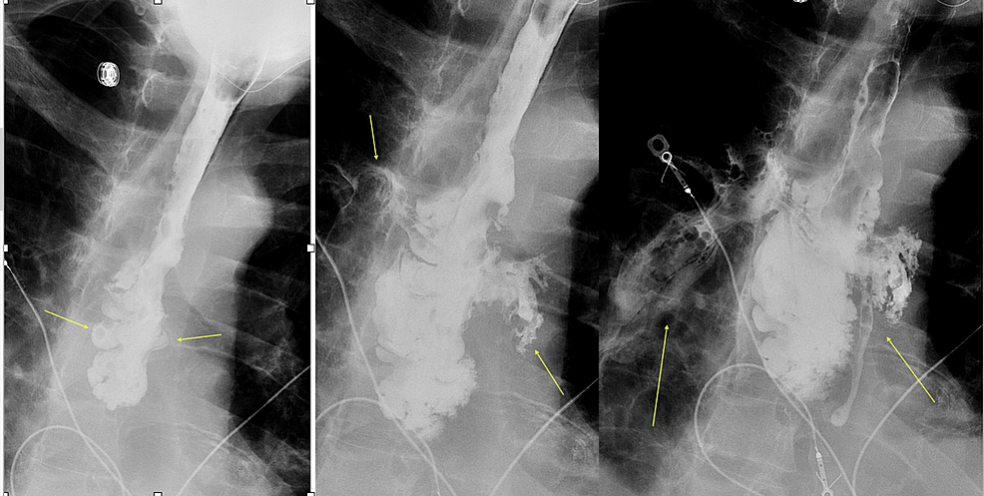
The initial esophagogram image demonstrates the passage of oral contrast into the mid esophagus with some irregularity (left image, arrows). Subsequent images show free extravasation of contrast beyond the expected confines of the esophageal lumen into the mediastinum consistent with an esophageal tear (arrows on middle and right images)

The patient was kept nil-by-mouth and started on intravenous (IV) meropenem, fluconazole, and decadron to treat mediastinitis and COVID pneumonia. He was not given Remdesivir due to his poor kidney function. An esophagogram (Figure 2) showed a free flow of oral contrast from the mid-esophageal level into the mediastinum consistent with esophageal rupture.

The patient had a thoracotomy and repair of the esophageal perforation with an intercostal muscle flap. We inserted a right chest tube and performed interventional radiology-guided placement of a gastro-jejunal tube. The patient further had an endoscopy and esophageal stent placement and bronchoscopy with lavage to remove the mucus plugging. Eventually, his symptoms improved, and the chest tube was removed. He completed a long course of IV meropenem and fluconazole. Multiple blood cultures remained negative throughout his hospitalization. He was discharged for subacute rehabilitation after 40 days of hospitalization.

## Discussion

The COVID-19 pandemic is a major health crisis that has severely drained medical resources. Researchers continue to investigate the nature of the virus and its health consequences. It usually presents with respiratory symptoms, but recently, gastrointestinal symptoms have also been reported [1-3]. In most hospital settings, clinicians are focused on managing COVID-19 respiratory symptoms. They also pay attention to their venous thromboembolism prophylaxis, given that the majority of patients with COVID pneumonia suffered from respiratory failure and extensive thrombosis [3].

Esophageal rupture is commonly due to iatrogenic causes such as upper gastrointestinal endoscopy, nasogastric tube insertion, caustic injury, and surgery. When transmural perforation occurs following forceful vomiting, it has been described as spontaneous perforation. It is also known as Boerhaave syndrome [4]. Dr. Hermann Boerhaave was the first to describe a case of esophageal perforation in 1724. An autopsy of his patient, Baron von Wassenaer, the Grand Admiral of Holland, revealed a transverse tear in the distal part of the esophagus. The pleural cavity was filled with gastric contents. The Grand Admiral had died after induced vomiting following a large meal [5-7].

Spontaneous perforation of the esophagus results from a sudden increase in intraesophageal pressure combined with negative intrathoracic pressure (e.g., severe straining or vomiting). Rupture of the intrathoracic esophagus results in contamination of the mediastinal cavity with gastric contents. This leads to chemical mediastinitis with mediastinal emphysema and inflammation. Subsequently, there is a bacterial infection and mediastinal necrosis. Rupture of the overlying pleura by mediastinal inflammation or by the initial perforation directly contaminates the pleural cavity, resulting in pleural effusion [4,7].

It is thought that only 50% of Boerhaave syndrome cases classically present with the Mackler triad of chest pain, vomiting, and subcutaneous emphysema due to esophageal rupture [4]. This leads to late diagnosis, delayed treatment, and increased mortality [6-7]. Therefore, diagnosis of Boerhaave syndrome with a patient with severe respiratory and gastrointestinal symptoms should not be delayed. Rapid implementation of the principal interventions, which includes sepsis control, adequate drainage, perforation repair, and antibiotic therapy to prevent death from sepsis and multiple organ failure, is critical [7].

In this case, we diagnosed the patient with Boerhaave syndrome, given his esophageal rupture attributed to his repetitive coughing and vomiting due to underlying COVID pneumonia. The proper management was started within the first 24 hours, which greatly impacted his clinical outcome.

## Conclusions

The early diagnosis of Boerhaave syndrome in our patient, and the prompt implementation of the principal management of sepsis control, adequate drainage, and surgical intervention, resulted in the patient’s good outcome. Despite the rare incidence of Boerhaave syndrome and the limited resources during the COVID-19 pandemic, the patient survived. Since we are living in a COVID-19 pandemic, we must be ready to diagnose and treat a wide variety of this disease’s complications and unusual presentations.
